# A Survey of Recent Synthetic Applications of 2,3-Dideoxy-Hex-2-enopyranosides

**DOI:** 10.3390/molecules20058357

**Published:** 2015-05-08

**Authors:** Ana M. Gomez, Fernando Lobo, Silvia Miranda, J. Cristobal Lopez

**Affiliations:** Instituto de Química Orgánica General (*IQOG-CSIC*), Juan de la Cierva 3, 28006 Madrid, Spain; E-Mails: fernando.lobo@iqog.csic.es (F.L.); s.miranda@csic.es (S.M.)

**Keywords:** hex-2,3-enopyranosides, cycloaddition, glycosylation, epoxidation, osmylation, Ferrier rearrangement, *de novo* synthesis

## Abstract

Unsaturated carbohydrate derivatives are useful intermediates in synthetic transformations leading to a variety of compounds. The aim of this review is to highlight the rich chemistry of ∆-2,3 unsaturated pyranosides, emphasizing the variety of transformations that have been carried out in these substrates during the last decade.

## 1. Introduction

**Scheme 1:**
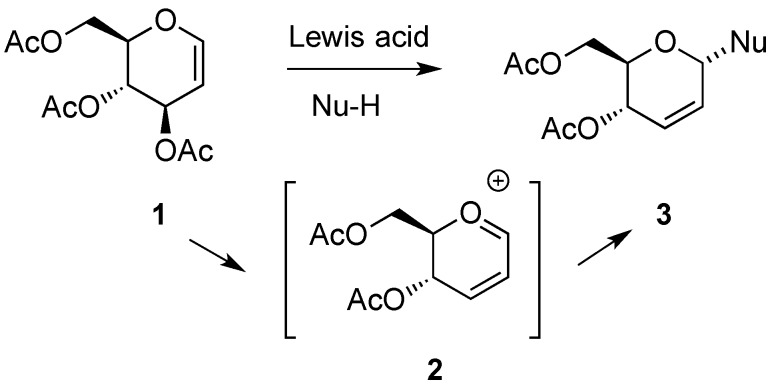
Ferrier rearrangement route to hex-2-enopyranoses **3**, from glucal **1**.

Hex-2-enopyranoses, e.g., **3**, also known as pseudoglycals, have provided fertile ground for synthetic and mechanistic developments in carbohydrate chemistry during the last decades [1,2]. The first report of a molecule belonging to this category was made by Fischer [3], although it was not until a decade later that its correct structure could be established by Bergmann [4]. However, the process **1**→**3** (Scheme 1), which made hex-2-enopyranosides broadly recognized synthetic intermediates, was only rendered available on a preparative scale in 1969 by Ferrier and Prasad [5]. This reaction has come to be known as the Ferrier I rearrangement, and the cationic intermediate **2** has since played a relevant role in many carbohydrate transformations [6,7]. From the outset, hex-2-enopyranosides have been employed in a plethora of synthetic endeavors [8,9,10]. Excellent coverage of the chemistry and synthetic applications of hex-2-enopyranosides has appeared regularly in the yearly issues of *Carbohydrate Chemistry, Specialist Periodical Reports*, until 2003 [11]. The vast contribution to the chemistry of hex-2-eno-pyranosides developed in the Fraser-Reid group, covering more than 20 years of research in the area, has recently been reviewed [12].

The aim of this review is to highlight synthetic transformations on 2,3-dideoxy-hex-2-eno-pyranosides reported during the last decade, 2003–2014.

## 2. Synthetic Routes to Hex-2-enopyranosides

The most widely used method for the preparation of 2,3-unsaturated hex-2-enopyranosides involves the Ferrier reaction, applied to glycal derivatives. Early studies on the Ferrier rearrangement made use of simple Lewis acids, e.g., BF_3_·Et_2_O, as promoters [5]. Since then, considerable attention has been devoted to the investigation of alternative catalysts for this transformation. In this context, a large number of publications involving the use of a variety of metallic, non-metallic, and heterogeneous catalysts have appeared. A report dealing with the promotors and nucleophiles currently used for the Ferrier rearrangement have been recently published, and readers in search of comprehensive information on this reaction are directed to it [13].

**Scheme 2:**
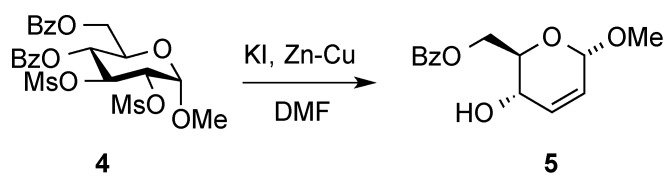
Fraser-Reid and Boctor’s route to hex-2-eno-pyranosides.

Besides the Ferrier rearrangement, outlined in Scheme 1, additional routes to access hex-2-enopyranoses from carbohydrates have also been described. Thus, Fraser-Reid and Boctor made use of the reductive elimination of vicinal disulfonates [14] to gain access to **5** (Scheme 2) [15].

**Scheme 3:**
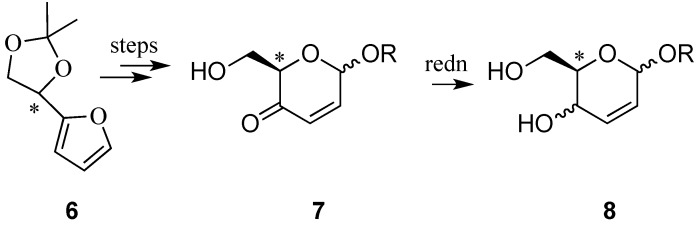
Achmatowicz and Zamojski’s *de novo* route to hex-2-enopyranosides from furylcarbinols.

A more circuitous route to allylic pyranosides from non-carbohydrate sources was developed by Zamojski and Achmatowicz (Scheme 3) [16,17]. In 1971, they reported the oxidative rearrangement of 2-furanylcarbinols into highly functionalized pyranones, e.g., **6**→**7**, to gain access to hex-2-eno-pyranosides **8** (Scheme 3). In the original Achmatowicz approach, the furfuryl carbinol is oxidized with bromine in the presence of methanol under weakly basic conditions. Many other modifications of the original Achmatowicz procedure, such as oxidation of the furan ring with *m*-CPBA [18], dimethyldioxirane [19], NBS [20,21], *tert*-BuOOH\VO(OAc)_2_ [22], or H_2_O_2_-titanium silicalite [23], have also been used for this transformation. This route has the advantage that the original configuration of the alcohol moiety in the furylcarbinol is preserved and, therefore, the method is amenable to the preparation of both d- and l-series [24,25,26].

**Scheme 4:**
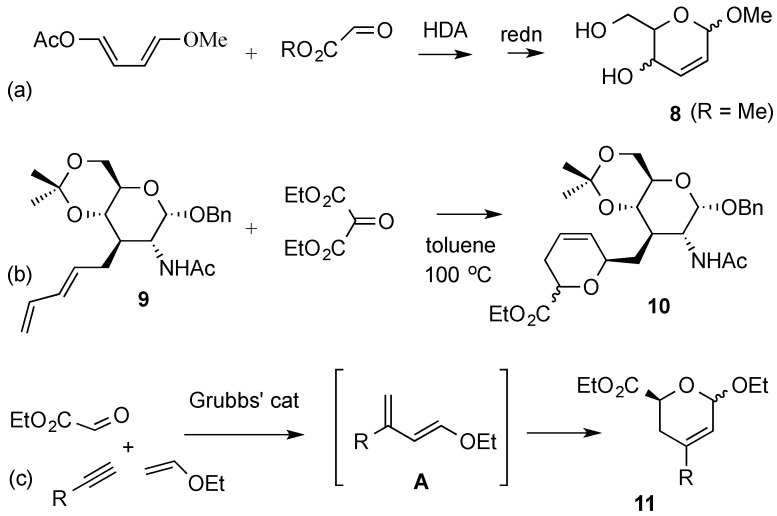
Hetero Diels-Alder (HDA) routes to hex-2-eno-pyranosides.

The hetero Diels-Alder reaction (HDA) has been amply used in the de novo synthesis of hexoses, and in many those instances 2,3-unsaturated derivatives have been key intermediates in these protocols [27,28,29]. Pioneering work by Danishefsky’s group had shown that hexoses could be accessed by Lewis acid-catalyzed HDA reaction of alkylated siloxy dienes with aldehydes via the intermediacy of labile 3-*O*-silyl-2,3-unsaturated glycoside adducts [30,31]. The hetero-Diels Alder reaction between substituted 1,4-dialkoxy-1,3-dienes and activated carbonyl compounds such as glyoxylates also provides access to hex-2-enopyranosides, e.g., **8**, from non-carbohydrate sources (Scheme 4a) [32,33]. This process can be promoted simply by heating, [33], by use of high pressure [33], or by Lewis acid catalysis [32]. A HDA reaction has been used to gain access to a pseudo C-disaccharide **10** from a d-glucosamine diene **9** (Scheme 4b) [34]. More recently, a one-pot multicomponent approach to 3-branched-2,3-unsaturated hexopyranoses **11** has been devised by Botta and co-workers (Scheme 4c) [35]. The protocol, in which a monosubstituted alkyne, ethyl vinyl ether and ethyl glyoxalate were combined, involved an enyne cross-metathesis (Grubb’s catalyst, 2nd generation) [36] leading a diene intermediate (**A**), followed by an *in situ* HAD reaction.

**Scheme 5:**
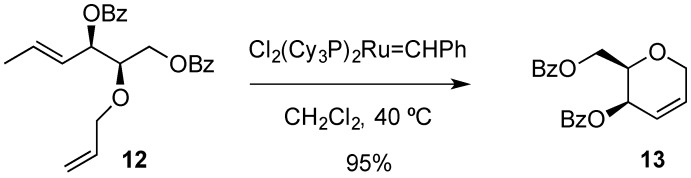
Ring-closing metathesis route to hex-2-eno-pyranose derivatives.

Ring-closure metathesis has become an important tool in organic synthesis and its application to carbohydrate chemistry [37] has included the synthesis of hex-2,3-enopyranose derivatives (Scheme 5). For example, dibenzoate **12** yielded 1-deoxy-hex-2,3-enopyranose **13** via ring-closing metathesis [38].

**Scheme 6:**
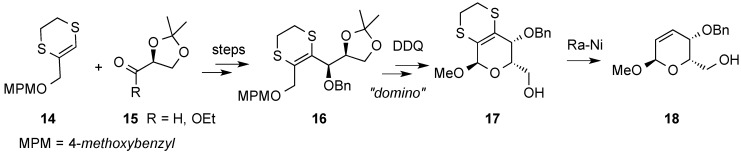
Guaragna’s group *de novo* approach to hex-2-enopyranosides.

An acid-catalyzed domino reaction has been developed by Guaragna and coworkers as a synthetic route to enantiomerically pure l-hex-2-enopyranosides (Scheme 6) [39]. Their strategy started from the three-carbon homologating agent **14**, prepared in a few steps from methyl pyruvate, and a chiral building block derived from l-glyceraldehyde **15**, which provides the inherent chirality at the C5 stereocenter of the final product, **18** [40]. The ring closure of the intermediate **16** was effected by a domino process triggered by DDQ in CH_2_Cl_2_/MeOH involving five steps: MPM protecting-group removal, oxidation of the ensuing primary alcohol, aldehyde dimethoxyacetalation, isopropylidene group cleavage, and ring closure. Finally, desulfuration of **17** with Raney-Ni led to 2,3-unsaturated-l-pyranoside **18**.

## 3. Reactions of Hex-2-enopyranosides

One of the reasons behind the ample use of hex-2-enopyranosides in carbohydrate chemistry might lie in their rich synthetic potential. They undergo standard alkene-addition reactions including hydrogenation, hydroxylation, oxyamination, or epoxidation, often with very high if not complete stereoselectivity. Incorporation of additional functionality that polarizes the alkene group, such as nitro or sulphonyl substituents, makes Michael-like additions possible, which take place with regiospecific introduction of nucleophiles. Hex-2-enopyranosides are also ideally structured to take part in sigmatropic rearrangements, the most straightforward of which involve compounds with allylic ester groups. Furthermore, the Δ^2,3^ insaturation in hex-2-enopyranosides confers a higher reactivity to both the anomeric (C-1) acetal and the C-4 hydroxyl group, opening new avenues for nucleophilic functionalization. Oxidative transformations are also of synthetic value since they might lead to unsaturated enones, unsaturated lactones, or to 6-formyl derivatives, depending on the conditions employed.

### 3.1. Addition Reactions

**Scheme 7:**
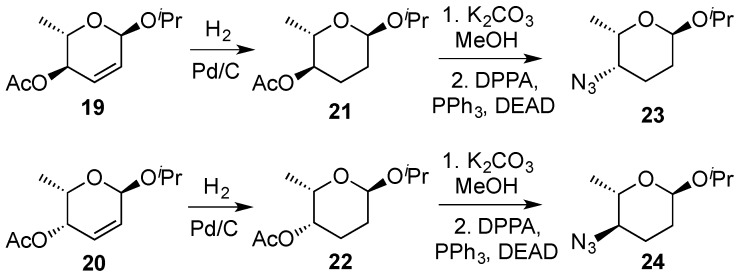
Zhang’s synthesis of 4-substituted uncommon-sugars.

Hydrogenation reactions of 2,3-enopyranosides have generated interest as a tool for delivering deoxy sugars which are present in biologically intriguing compounds [41,42]. For example, it has been shown that in aminoglycosides, the removal of hydroxyl goups imparts *in vitro* stability by lessening the abilities of naturally occurring glycosidase enzymes to degrade the structure [43]. In this context, Zhang *et al.* developed a divergent strategy for constructing uncommon l-sugars with 4-substitution. They employed 2,3-eno-pyranosides **19** and **20** and a combination of typical palladium on carbon hydrogenation and Mitsunobu reactions involving the use of diphenylphosphorylazide (DPPA) (Scheme 7) [44].

**Scheme 8:**
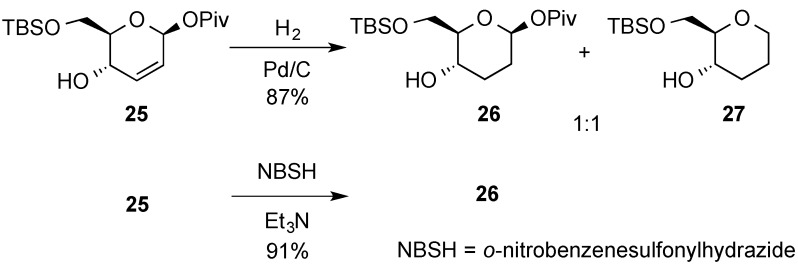
O’Doherty’s diimide reduction of 2,3-enopyranosides.

O’Doherty’s goup proposed a diimide reduction as an alternative to the direct hydrogenation reaction of 2,3-enopyranosides where partial hydrogenolysis could compete. The method was applied to allyl alcohol **25** that upon standard hydrogenation conditions produced a significant amount of the hydrogenolysis product **27** (Scheme 8). Thus, by exposing allylic alcohol **25** to an excess of *o*-nitrobenzenesulfonyl hydrazide (NBSH) and Et_3_N, an excellent yield of the desired pivalate **26** could be obtained [45].

**Scheme 9:**
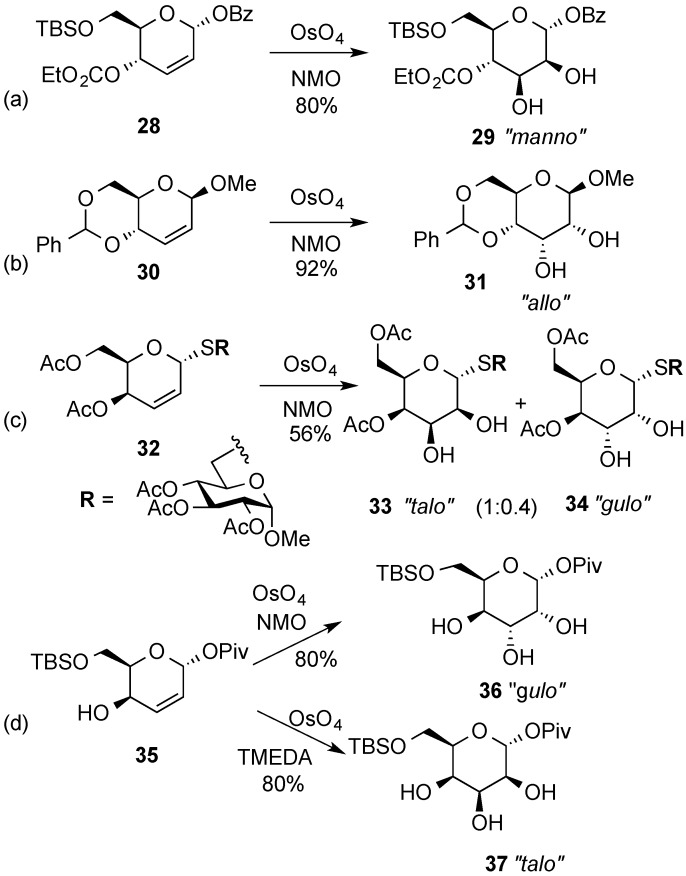
*Cis*-dihydroxylation of hex-2,3-enopyranosides by OsO_4_.

*Cis*-hydroxylation of the double bond in hex-2,3-enopyranosides under common conditions (OsO_4_, H_2_O_2_ or RuCl_3_/NaIO_4_) normally occurs from the sterically more accesible face of the sugar ring in a process that is very often stereospecific. For example, the dihydroxylation reaction of 2,3-dideoxy-α-d-erythro hex-2-enopyranoside **28**, where both the anomeric substituent and the 4-substituent are located below the ring, occurs exclusively from the upper face of the molecule, resulting in formation of α-d-mannopyranoside **29** (Scheme 9a) [26]. However, osmylation of β-d-erythro-2-enopyranoside **30**, where the C-1 and C-4 substituents are disposed in opposite faces of the pyranose, led exclusively to β-d-allopyranoside **31**, with the osmium approach taking place anti- to the anomeric substituent (Scheme 9b) [46]. Similarly, dihydroxylation of galactal derivative **32** occurred mostly from the β-face opposite to the anomeric substituent leading to “talo”·derivative **33**, although some “gulo” derivative **34** was also obtained (Scheme 9c) [47]. On the other hand, exposure of allylic alcohol **35** to OsO_4_/NMO in *t*-BuOH/H_2_O afforded gulose isomer **36** in 80% yield, whereas the protected talose isomer **37** was selectively produced upon treatment of **35** with the TMEDA adduct of OsO_4_ (Scheme 9d) [26].

**Scheme 10:**
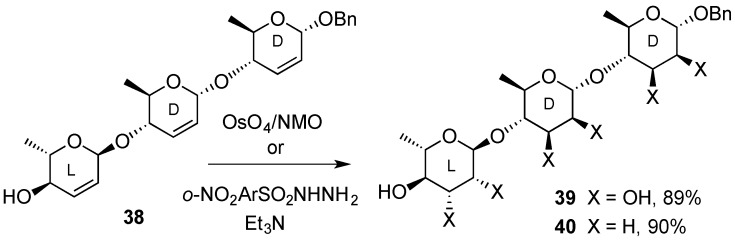
O’Doherty’s synthesis of 1,4-linked α-rhamno-trisaccharides.

This methodology has been used by O’Doherty and coworkers in a highly efficient *de novo* route to various oligosaccharide motifs containing both d- and l-sugars [48]. For example, osmium-catalyzed dihydroxylation of tri-2,3-enopyranoside derivative **38** afforded the 1,4-linked α-rhamno-pyranose **39**, while the global reduction of the double bonds with excess diimide provided 2,3-dideoxy oligosaccharide **40** in excellent yield (Scheme 10).

**Scheme 11:**
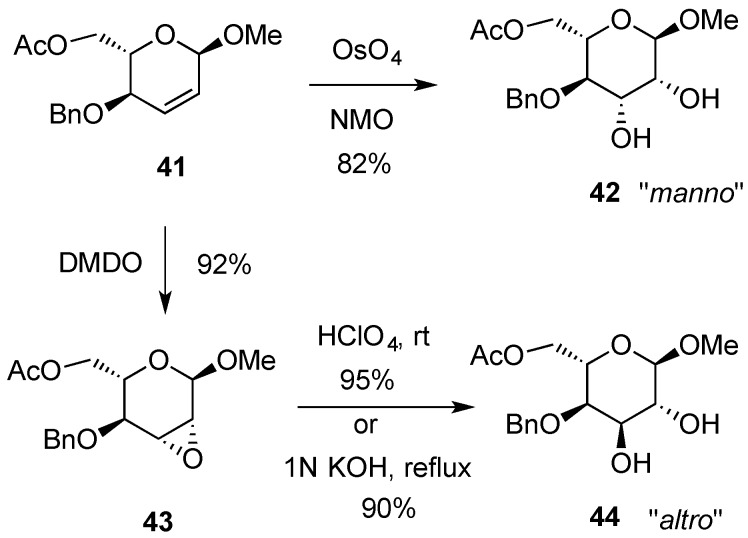
Alternative routes for *cis*- and *trans*-dihydroxylation of hex-2,3-enopyranoside **41**.

Dihydroxylation products can also be obtained by sequential epoxidation/ring-opening reactions. In these substrates, the stereochemistry of the epoxidation is highly influenced by the nature of the allylic hydroxyl groups. In general, free hydroxyl groups direct the approach of the incoming oxygen atoms to the double bond in a *syn* manner, whereas an *anti*-approach is observed when the hydroxyl groups are protected [49]. Ring-opening of epoxides arising from hex-2,3-enopyranosides tend to form trans-diaxial products, due to the Fürst-Platnner rule [50] and therefore this approach is complementary to the previously mentioned *cis*-hydroxylation. For instance, hex-2-enopyranoside **41** under common Upjohn conditions gave exclusively methyl l-mannopyranoside **42**, whereas l-altropyranoside **44** was obtained after treatment with dimethyldioxirane and the subsequent ring opening of the 2,3-anhydro derivative **43** by acid or by base-catalyzed hydrolysis (Scheme 11) [40].

**Scheme 12:**
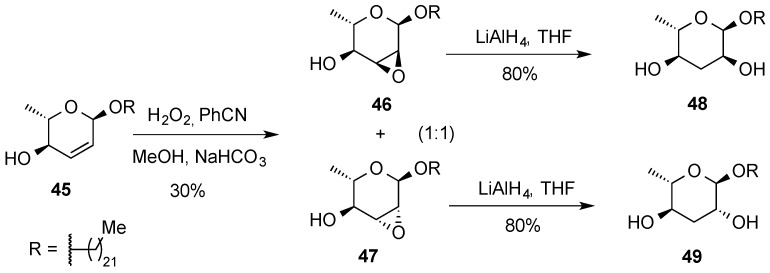
Synthesis of ascarosides **48** and **49**.

Joly *et al.* [51] found that the double bond of l-sugar derivative **45** failed to react with MCPBA. However, when the reaction was performed under the conditions of Payne (H_2_O_2_/PhCN), a mixture of epoxides **46** and **47** was formed. The long aglycone chain is likely hindering the attack on the α-side of the 2,3-enopyranoside and lowering the overall yield as well. The epoxides were then reductively ring-opened by LiAlH_4_ to form ascaroside models **48** and **49** (Scheme 12).

**Scheme 13:**
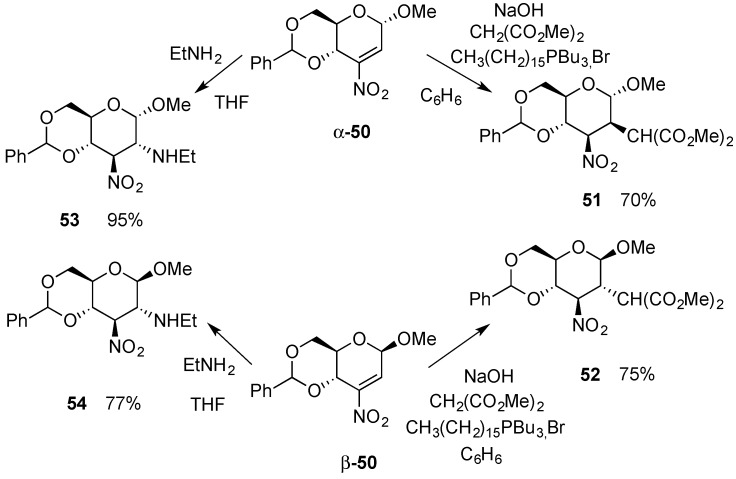
Michael addition on isomeric 3-nitro-hex-2-enopyranosides **50**.

The incorporation of chemical functionality that polarizes the alkene on 2,3-enopyranosides makes possible Michael-like additions resulting in the regioselective introduction of nucleophiles. Several examples of Michael reactions on 3-nitro-hex-2-enopyranosides, e.g., **50**, were previously reported by Sakakibara’s group. In these reactions, active methylene compounds [52,53,54] and sterically demanding purine bases [55] reacted regio- and stereoselectively at C-2 from the side opposite to the anomeric substituent (e.g., **51** from α-**50** and **52** from β-**50**) (Scheme 13). Amines, however, produced thermodynamically more stable C-2 equatorial products (**53** and **54**) irrespective of the anomeric configuration of the starting glycoside [56]. These results have been discussed in terms of electrostatic interactions [57], stereoelectronic control [57], steric hindrance [57], A-strain [58] and also hydrogen bonding [58]. Dideoxy-hex-2-en-4-ulopyranosides, on the other hand, always produced epimeric mixtures at C-2 [59,60,61].

**Figure 1:**
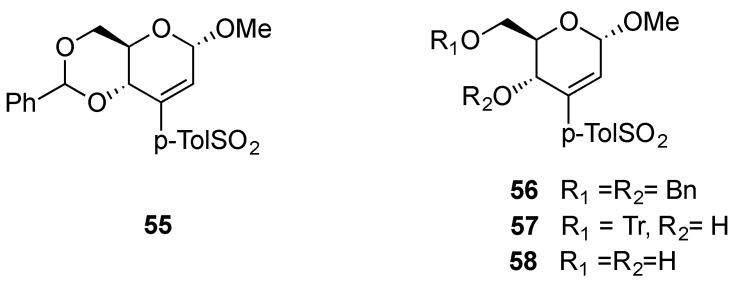
Vinyl-sulfone Michael acceptors **55**–**58**.

More recently, Pathak and coworkers have studied the behavior of vinyl sulfone-modified hex-2-enopyranosides. Michael additions, followed by desulfonylation with Na-Hg (6 mol-%) of the resulting adducts [62], allowed the regio- and stereo-selective introduction of nucleophiles in 2,3-eno-pyranosides. They found a remarkable influence of the protecting groups of the hydroxyl moieties on the reaction patterns [63]. For example, although phenylmethylene-protected vinyl sulfone **55** reacts with both primary and secondary amines in a Michael-fashion, only primary amines react with the dibenzyl-protected, *O*-trityl protected or unprotected derivatives **56**, **57** and **58** respectively (Figure 1).

**Scheme 14:**
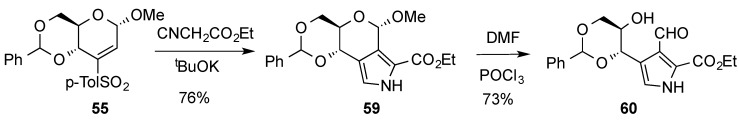
Synthesis of functionalized chiral pyrrol **60** from vinylsulfone **55**.

This strategy has amply been employed by Pathak’s group in the synthesis of a variety of compounds including aminosugars [64], branched-chain sugars [65], isonucleosides [62], and chiral pyrroles [66]. For example, conjugate addition of the anion generated from ethyl isocyanoacetate to vinylsulfone **55** afforded a pyrrole derivative **59**, which by subsequent treatment with POCl_3_/DMF afforded chiral pyrrole **60** (Scheme 14).

**Scheme 15:**
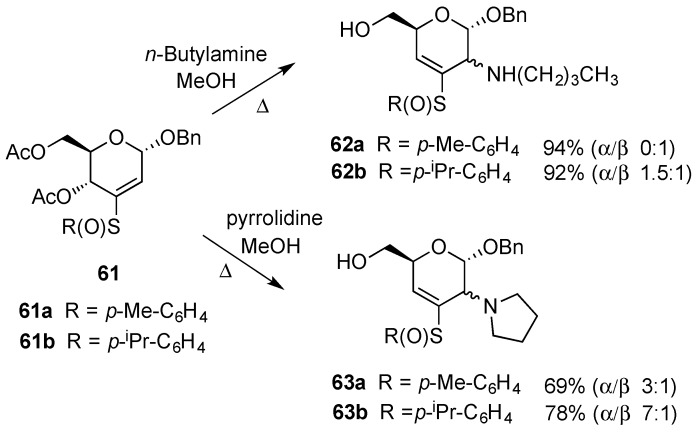
Michael addition on 3-arylsulfinyl-hex-2-enopyranosides **61**.

On the other hand, 2,3-unsaturated 3-arylsulfinyl pyranosides have been shown to undergo nucleophilic additions at C-2 with facial selectivities that are influenced by the nucleophile and the substituent on the sulfinyl sulphur [67]. For example, the reaction of **61a** with primary amines (carbon and sulphur nucleophiles were also used) led to adduct **62a**, with the addition of the nucleophile preferring an axial orientation at C-2 and with concomitant elimination of acetic acid to form an allylic bond at ∆^3,4^. Conversely, the related reaction of **61** with a secondary amine led to a mixture of epimeric 2-deoxy-2-amino compounds **63a** where the major product displayed a C-2 equatorial orientation. Furthermore, the influence of the α-sulfinyl substituent on the stereochemical outcome of the reaction also became clear. Thus, reaction of sterically congested (*p*-isopropylphenyl)vinyl sulfoxide **61b** with pyrrolidine produced a C-2 α/β 7:1 epimeric mixture, whereas reaction of pyrrolidine with *p*-tolyl vinyl sulfoxide **61a** produced a C-2 α/β 3:1 epimeric mixture (Scheme 15). A similar trend was also observed in the reaction of **61a** and **61b** with primary amines, leading to **62a** and **62b**, respectively (Scheme 15).

Finally, hex-2-enopyranosides have shown to be popular starting materials in the preparation of biologically relevant 2,3-dideoxy-3-amino sugars in which the amino group is *cis* to a vicinal (C4-OH) hydroxyl group [68]. Thus, Fraser-Reid’s group introduced the iodine mediated cyclization of (C-4) allylic imidates to the Δ^2,3^ unsaturation on hex-2-enopyranosides, which directed the *cis* entry of the nitrogen function [69,70,71]. Several other functionalities such allylic carbamates or isoureas have been used since in this electrophile induced cyclization. Hydrolysis of the resulting oxazoline paves the way to the desired *cis* amino alcohol functionality [68]. In this context, Takahashi and co-workers have reported the synthesis of l-vancosamine, l-ristosamine, l-saccharosamine, and l-daunosamine by use of an electrophile-induced [*o*-iodoxybenzoic acid (IBX)] [72] cyclization of allylic carbamates [73].

### 3.2. Nucleophilic Substitutions

**Scheme 16:**
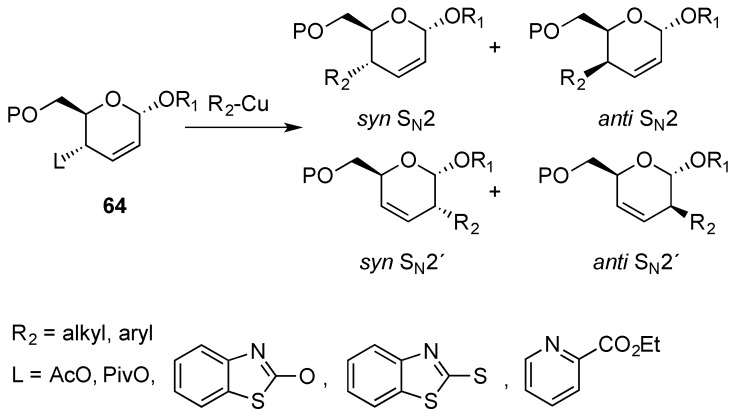
Allylic substitution reaction on 2,3-enopyranoside **64** and possible regio- and stereoisomers.

Reactions that allow the displacement of the C-4 allylic group on 2,3-enopyranosides also open opportunities for functionalization. Early reports were based on the nucleophilic allylic substitution with copper reagents. This possibility was limited to substrates containing acetoxy and pivaloxy, leaving groups to afford *anti* S_N_2' products in moderate to good yields (Scheme 16) [74,75,76]. In contrast, reaction of the corresponding benzothiazolyl thio ethers afforded *syn* S_N_2' adducts [77,78]. More recently, allylic substitution of substrates possesing the picolinoxy group have been studied and it was found that different alkyl and aryl groups could easily be installed on the pyran ring with anti S_N_2' selectivity [79].

**Scheme 17:**
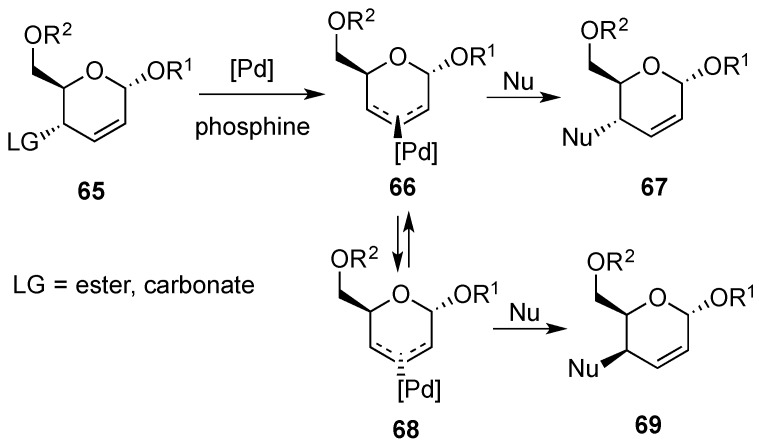
Palladium-mediated allylic substitution.

Of particular relevance is the Pd-catalyzed substitution of allylic esters or carbonates by carbon and nitrogen nucleophiles [80,81,82,83,84,85]. A mechanistic picture of this process is displayed in Scheme 17. Even though one or more of these paths may become competitive, the use of more reactive allylic carbonates usually prevents the presence of any palladium(0) complex (**66**) in solution, and a remarkable selectivity is observed. The net overall retention in the palladium-mediated nucleophilic addition is then attributed to retention of stereochemical integrity during both generation of the π-allyl-Pd intermediate and the subsequent addition of the nucleophile.

**Scheme 18:**
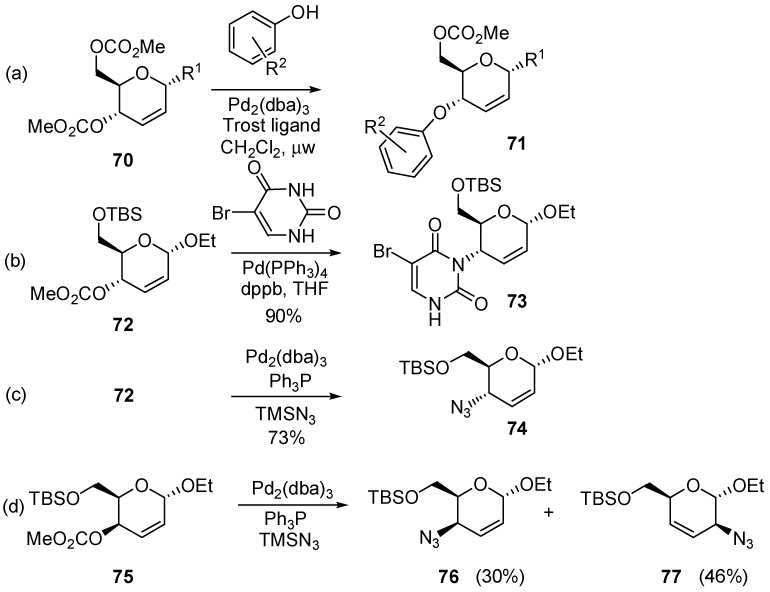
Examples of palladium-mediated allylic substitution.

This methodology has been applied to the addition of phenols [86], heterocyclic nucleophiles including uracil derivatives [87], and/or azides [88,89]. Nucleophilic substitution carried out in alkyl α-d-*erythro*-hex-2- enopyranosides, e.g., **70** and **72**, took place with a very high regio- and stereoselectivity to provide C-4 substituted derivatives **71** and **73**, respectively (Scheme 18a,b). Likewise, the palladium-catalyzed reaction of **72** with TMSN_3_ led regio- and stereoselectively to 4-deoxy-4-azido derivative **74** (Scheme 18c). On the other hand, palladium-catalyzed reaction of the epimeric 2,3-dideoxy-α-d-*threo*-hex-2-enopyranoside **75**, with TMSN_3_ provided a regioisomeric mixture of **76** and **77** arising from attack at positions C-4 and C-2 of the π-allyl complex (Scheme 18d).

### 3.3. [3,3]-Sigmatropic Rearrangements

**Scheme 19:**
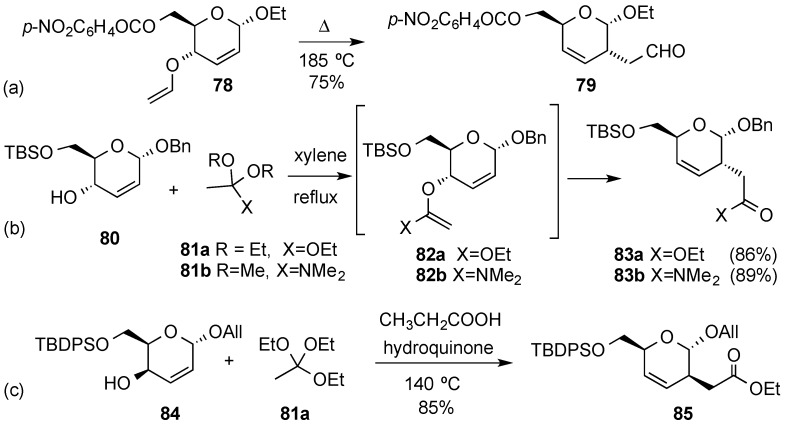
Examples of Claisen rearrangements.

Unsaturated sugar derivatives are ideally structured to take part in [3,3]-sigmatropic rearrangements that allow the construction of carbón-carbon or carbón-heteroatom bonds. For example, in 1973 Ferrier *et al.* showed that 4-vinyl 2,3-enopyranoside **78** could undergo a Claisen rearrangement upon heating at 185 °C to give the branched-chain aldehyde **79** (Scheme 19a) [90]. The reaction took place readily and in a completely stereoselective manner, as was expected for such suprafacial allyl rearrangement. However, the yield in the mercury-catalyzed preparation of the required vinyl derivative **78** was low (30%). As a synthetic alternative, Krohn *et al.* described the reaction of the related allylic alcohol **80** with an eightfold excess of orthoacetic ester **81a** in the presence of catalytic amounts of propionic acid to afford the corresponding ester **83a** in good yield as one single isomer (Scheme 19b) [91]. Similarly, the Eschenmoser variant of the Claisen rearrangement allowed access to **83b** (89% yield) from allylic alcohol **80** by using 1.5 equiv. of *N,N*-dimethylacetamide dimethyl diacetal **81b** [91]. The C-4 epimeric allylic alcohol **84**, also experienced a Claisen-Johnson rearrangement in a completely stereoselective manner leading to C-2 branched derivative **85**, in good yield (Scheme 19c) [92].

**Scheme 20:**
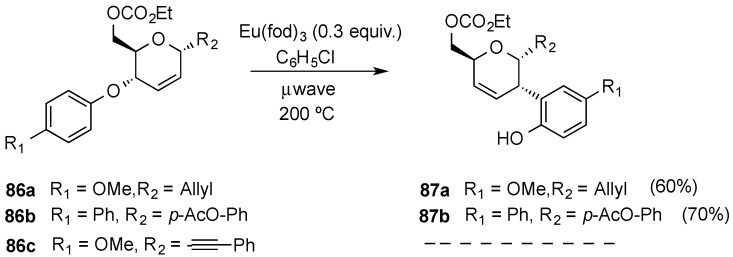
Sigmatropic rearrangement of allyl phenyl ethers.

Porco and coworkers evaluated Eu(III)-catalyzed Claisen rearrangement of allyl phenyl ethers derived from hex-2,3-enopyranosides (Scheme 20). The reaction required microwave heating at elevated temperatures (200 °C). Representative allyl and aryl *C*-glycosides **86a**–**b** underwent [3,3]-sigmatropic rearrangement to provide phenols **87a**–**b** (Scheme 20). However, preliminary studies had demonstrated that this reaction depends on the aglycone substituent as alkynyl *C*-glycoside **86c** did not readily undergo rearrangement, even after prolonged heating with excess Eu(fod)_3_ [86].

**Scheme 21:**
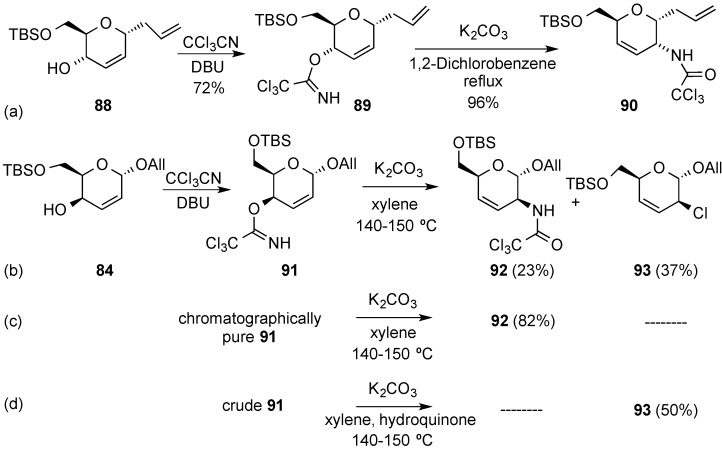
Examples of Overman rearrangements.

Related Overman [3,3]-sigmatropic rearrangements have also been used to incorporate amine functions at C-2 position in hex-2,3-enopyranosides. Thus, allylic trichloroacetimidate **89**, readily obtained from *C*-allyl glycoside **88**, allowed the efficient installation of a secondary amine at C-2 in compound **90** upon reflux in 1,2-dichlorobenzene in the presence of K_2_CO_3_ [93]. The analogous reaction with a related epimeric alcohol in allyl glycoside **84** required considerable experimentation, though, since the expected amide **92** was obtained alongside a chlorinated side-product **93** [94]. It was subsequently found that the formation of allylic chloride **93** was related to the degree of purity of trichloroacetimidate **91** used in the rearrangement. Thus, chromatographically pure imidate **91** underwent the Overman rearrangement to give the expected amide **92** in 82% yield (Scheme 21c). However, when the rearrangement was carried out with non-purified trichloroacetimidate **91**, and in the presence of hydroquinone as a radical scavenger, the synthetically useful chloride **93** could obtained as the single product in a moderate yield (Scheme 21d) [95].

### 3.4. Oxidative Transformations

**Scheme 22:**
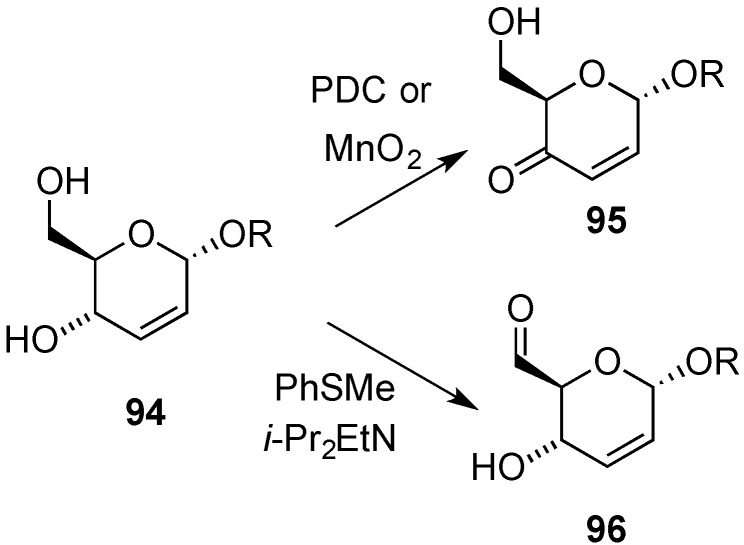
Chemoselective oxidation of 2,3-dideoxy-α-d-erythro-hex-2-eno-pyranoside **94**.

Oxidative transformations of hex-2,3-enopyranosides are also of synthetic value since they might lead to unsaturated enones, unsaturated lactones, or 6-formyl derivatives, depending on the conditions employed. Oxidation of diols **94** can be attained regioselectively at either *O*-4, or *O*-6, to give enones, e.g., **95** [96,97,98,99,100,101], or aldehydes, e.g., **96** [102], respectively (Scheme 22). For example, ethyl 2,3-dideoxy-α-d-erythro-hex-2-enopyranoside (**94**, R=Et) undergoes chemoselective allylic oxidation upon treatment with manganese dioxide or pyridinium dichromate to give hex-2-enopyranoside-4-ulose **95**, whereas selective oxidation of the primary hydroxyl group can be effected by a modification of the Corey-Kim procedure [103], as recommended by Fraser-Reid and co-workers, leading to aldehyde **96** [104,105,106]. The latter compound (R = Et) was used in a stereoselective synthetic approach to (+)-asperlin [107].

On the other hand, synthetically useful 2,3-dideoxyhex-2-enono-1,5-lactones **99** can be accessed from hex-2-enopyranosides, e.g., **97**, through oxidation (30% H_2_O_2_, MoO_3_). This process, reported by Zamojski’s group, involved dehydration of initially formed allylic hydroperoxides, e.g., **98** (Scheme 5) [108,109]. However, more concise routes to lactones **99** involve the direct oxidation of the corresponding glycals, and in this context the methods described by Lichtentahler’s (mCPBA, BF_3_.Et_2_O) and Sinaÿ’s (PCC) groups are worthy of mention [110,111].

### 3.5. Cycloaddition Reactions

**Scheme 23:**
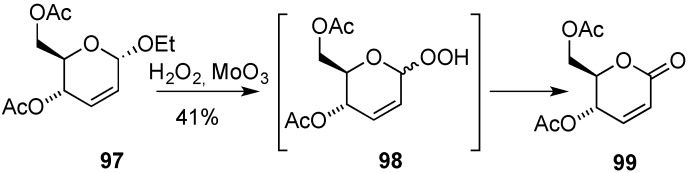
Zamojski’s route to unsaturated lactones from hex-2-enopyranosides.

**Figure 2:**
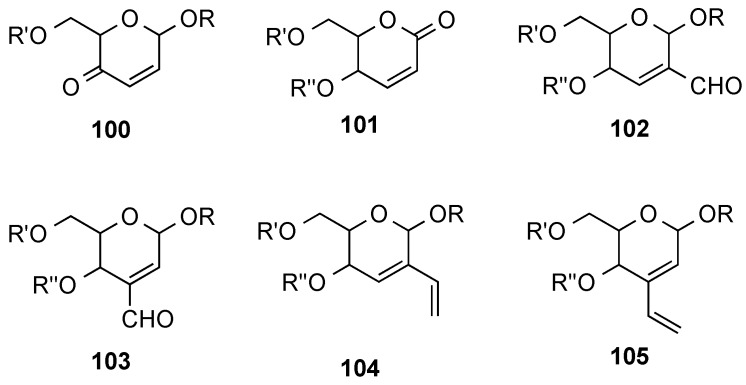
**∆**^2,3^**-**Unsaturated derivatives employed in cycloaddition reactions.

In order to participate in cycloaddition processes, the double bond in hex-2-eno-pyranoses has been incorporated into a variety of systems. For instance, oxidized derivatives such as enones **100** (related to **95**, Scheme 22) and unsaturated δ-lactones **101** (related to **99**, Scheme 23) were used in Diels-Alder [9] and dipolar cycloadditions [112,113,114,115] (Figure 2). Homologated derivatives such as isomeric enals **102** and **103** also found use as dienophiles [116] and heterodienophiles [117], and finally isomeric dienes **104** and **105** were reported to undergo stereoselective Diels-Alder reactions with maleic anhydride and dimethyl acetylenedicarboxylate, among other dienophiles [116,118].

During the last decade, Chmielewski’s group has continued its investigation on 1,3-dipolar cycloaddition of nitrones to carbohydrate derived δ-lactones, e.g., **101**. Their research has proven useful from theoretical and practical standpoints, and some of the resulting cycloaddition adducts have been applied to the synthesis of biologically relevant iminosugars [119].

**Figure 3:**
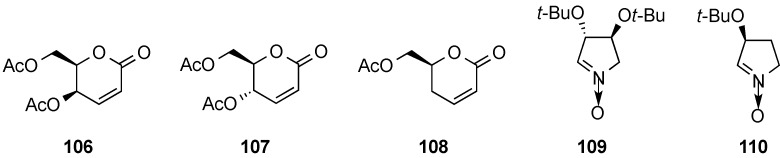
δ-Unsaturated lactones and chiral nitrones.

They have reported that the cycloaddition between aldono-1,5-lactones **106**–**108** and chiral five-membered cyclic nitrones **109** and **110** proceeded exclusively in the *exo* mode, to provide in many instances a single adduct as a result of double asymmetric induction (Figure 3) [120].

**Scheme 24:**
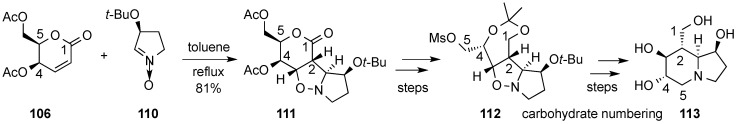
Chmielewski’s synthesis of 8-homocastanospermine **113**.

In particular, the cycloaddition reaction between lactone **106** and nitrone **110** resulted in the completely stereoselective formation of tricyclic derivative **111** as a consequence of an *exo*-approach of the nitrone and the *anti* addition to both the acetoxymethyl- and the 4-acetoxy group of the lactone (Scheme 24). The latter was then used in the synthesis of 8-homocastanospermine **113**, via key-intermediate **112**, which after cleavage of the isopropylidene group and hydrogenolysis of the N-O bond underwent intramolecular alkylation of the nitrogen atom, leading ultimately to **113** [121].

**Scheme 25:**
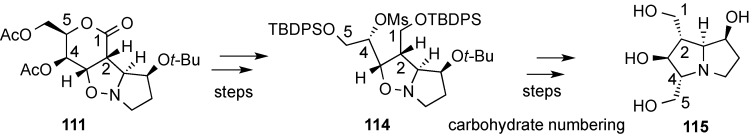
Chmielewski’s synthesis of 1-homoaustraline **115**.

Tricyclic adduct **111** was also employed in a related synthesis of a C-1 homologue of australine, 1-homoaustraline **115** (Scheme 25) [122]. Thus, chemistry related to the one mentioned-above when performed on mesylate **114** paved the way to 1-homoaustraline **115**.

**Scheme 26:**
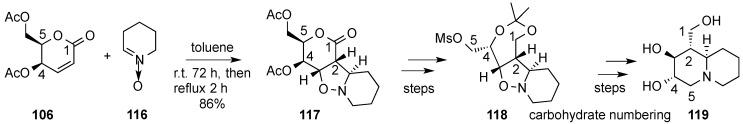
Chmielewski’s synthesis of 1-epilupinine **119**.

1,3-Dipolar cycloaddition of six-membered nitrone **116** with lactone **106** gave one single adduct, **117**, as the result of the *exo-anti* approach to both substituents of the lactone dipolarophile (Scheme 26) [123]. The latter was next transformed through a series of synthetic steps into mesylate **117** that led to 2,3-dihydroxy-epilupinine **119**.

The syntheses described above have benefitted from two key issues: (i) the high stereoselectivity of cycloadditions of simple nitrones to *threo*-lactones, e.g., **106**, when compared to *erythro*-lactones, e.g., **107**, and (ii) the easy rearrangement of the δ-lactone fragment in the adduct to a γ-lactone whose terminal diol could easily be cleaved.

**Scheme 27:**
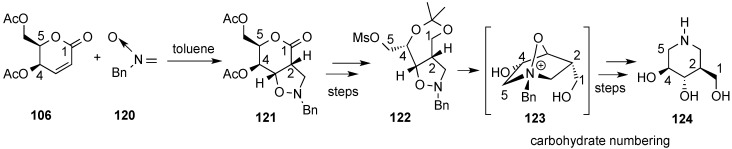
Chmielewski’s synthesis of (−)-isofagomine **124**.

Along this line, the cycloaddition of acyclic nitrones to carbohydrate-lactones was also studied for the preparation of iminosugars (or azasugars) [124] In this context, cycloaddition of nitrone **120** and lactone **106** produce one single adduct, **121**, which was processed by rearrangement to a γ-lactone and removal of the terminal (C-6) hydroxymethyl group into mesyl derivative **122** (Scheme 27). Deprotection of the hydroxy groups in the latter caused immediate intramolecular alkylation of the nitrogen atom leading to ammonium salt **123**. Finally, hydrogenolysis of **123** followed by several acetylation/deacetylation processes and hydrogenolysis of the *N*-benzyl substituent afforded (−)-isofagomine **124** [125].

Recent work by Chmielewski’s group has shown that the 1,3-dipolar cycloaddition to α,β-unsaturated δ-lactones is under kinetic control. Conversely, when γ-lactones are involved in the cycloaddition, the process becomes reversible and could be used to obtain the more stable, thermodynamic products. Owing to these properties along with the above-shown high stereoselectivity of their cycloaddition, δ-lactones have been employed for the kinetic resolution of racemic nitrones [126].

**Scheme 28:**
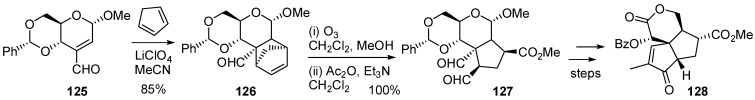
Synthetic approach to pentalenolactone **128**.

Testero and Spanevello reported a concise synthetic route to pentanelactone **128** from α,β-unsaturated aldehyde **125** (Scheme 28) [127]. The successful approach was based on two key steps. First, a completely stereoselective Diels-Alder cycloaddition of cyclopentadiene and enal **125** [128], with the diene approaching the dienophile from the β-face in an *exo*-mode of addition. Second, the ozonolysis of **126** took place via a completely regioselective cleavage leading to dialdehyde **127**. The latter was then transformed in ten steps into pentalenolactone **128**.

Further studies by this group have addressed the issue of the fragmentation of the primary ozonide in carbohydrate-derived norbornene systems. They showed that, in participating solvents, the remote substitution is responsible for the regioselective fragmentation of the intermediate ozonide [129,130,131].

**Scheme 29:**
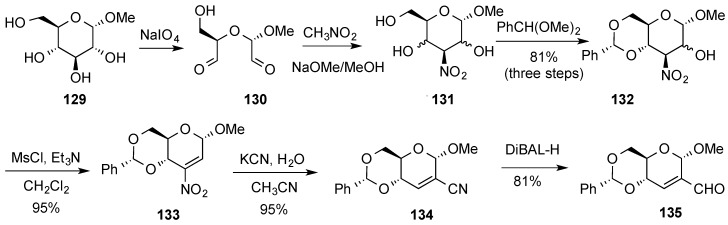
Spanevello’s synthetic route to enal **135**.

A multistep route to previously described enal **135** [128], has also been described (Scheme 29) [132]. The synthesis started with the oxidative cleavage of methyl-α-d-glucopyranoside **129** leading to dialdehyde **130**. Treatment of **130** with nitromethane in basic medium led to a mixture of 3-deoxy-3-nitro derivatives **131**. Benzylidenation of these derivatives followed by purification of the crude reaction provided epimeric mixture of alcohols **132** that upon elimination, mediated by treatment with MsCl and Et_3_N, yielded unsaturated nitro derivative **133**. Further processing of **133** via cyano derivative **134** permitted access to **135**.

**Scheme 30:**
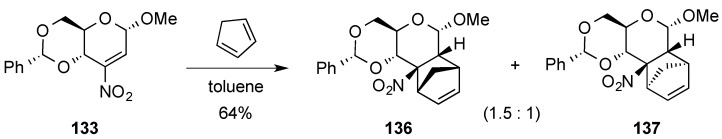
Diels-Alder cycloaddition between nitroalkene **133** and cyclopentadiene.

Finally, the thermal Diels-Alder reaction between sugar-derived nitroalkene **133** and cyclopentadiene yielded a mixture of *exo*- and *endo*- adducts **136** and **137**, respectively, where unlike previous examples, an α-facial selectivity in the approach of the diene was observed (Scheme 30) [133].

### 3.6. Glycosylation Reactions

The study of glycosylation reactions of 2,3-unsaturated hexenopyranoses has recently been addressed by mediation of either palladium or Lewis acid catalysis.

#### 3.6.1. Palladium Mediated Glycosylation

Feringa and O’Doherty’s groups addressed the issue of glycosylation with 2,3-unsaturated hexoses functioning as glycosyl donors [134].

**Scheme 31:**
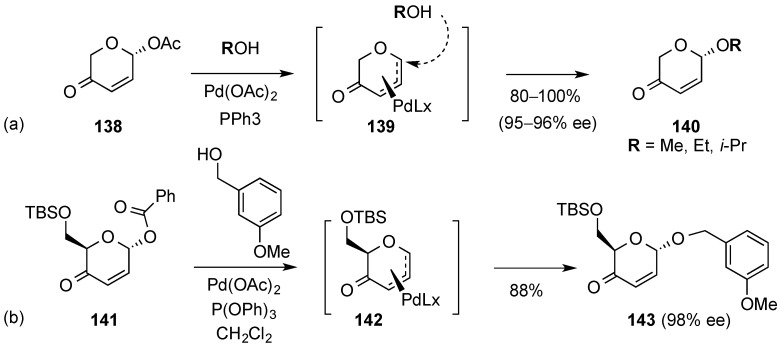
Feringa and co-workers’ palladium-catalyzed allylic substitution.

Following previous studies on palladium catalyzed allylic substitution on 6-acetoxy-2*H*-pyran-3(6*H*)-ones by alcohols (Scheme 31a) [135], Feringa and co-workers reported the stereoselective palladium catalyzed glycosylation of pyranones (Scheme 31b) [136]. The method proved to be particularly useful in synthesis since retention of stereochemistry at the allylic acetal moiety was observed in the newly formed glycosidic bond.

**Scheme 32:**
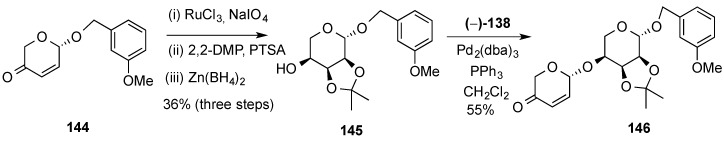
Feringa’s approach to iterative saccharide synthesis.

They next explored the feasibility of an iterative protocol based in this chemistry for saccharide synthesis. Thus, diastereoselective catalytic *cis*-dihydroxylation of **144** followed by acetonide formation on the ensuing diol and reduction of the ketone moiety paved the way to β-l-ribose derivative **145** (Scheme 32). This sugar was next glycosylated with (−)-**138** under palladium catalysis [Pd_2_(dba)_3_, PPh_3_] to give disaccharide precursor **146**.

**Scheme 33:**
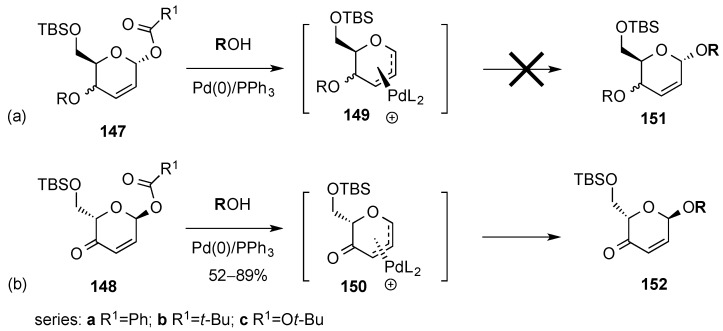
O’Doherty’s Pd-catalyzed glycosylation with pyranone donors **148**.

Shortly after Feringa’s findings, O’Doherty’s group reported on a similar transformation [137]. They studied the behavior of Pd π-allyl intermediates **149** and **150** arising from allylic alcohols **147** or unsaturated ketone **148**, respectively, and found that whereas reaction of **147** failed to provide any unsaturated glycoside **151** (Scheme 33a), π-allyl intermediate **150** reacted with a variety of alcohols to give allylic glycosides **152** in moderate to excellent yields (Scheme 33b). O’Doherty’s group ascribed these contrasting results to the higher electrophilicity of Pd π-allyl intermediate **150** compared to **149**. In this context, Lee and co-workers reported that the reaction of intermediates type **149**, generated from glycals rather than from hex-2,3-enopyranosides, with alcohol acceptors to give *O*-glycosylation products, e.g., **151**, could be carried out by activating the acceptor via zinc(II) alkoxide formation [138,139].

Further experimentation by O’Doherty’s group led to the use of *tert*-butyl carbonates **148c** as the preferred glycosyl donors. Their explanation for the improved reactivity of **148c** *versus* **148a**,**b**, was that *t*-BuOH and CO_2_, rather than carboxylic acids, were generated as leaving groups.

**Scheme 34:**
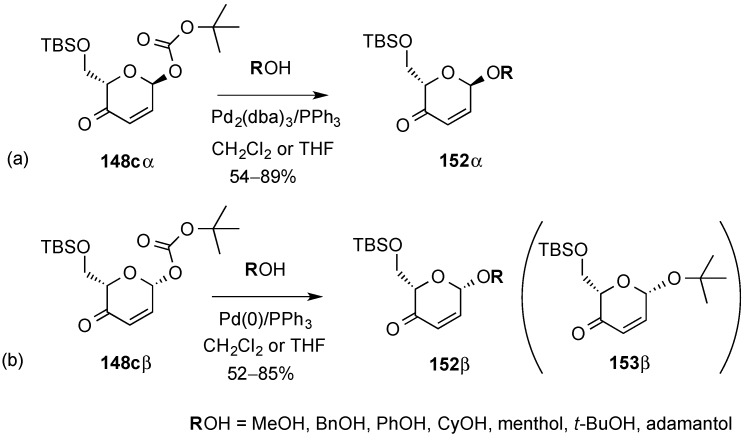
Pd-catalyzed stereoselective glycosylation.

The Pd-catalyzed glycosylation reaction proceeded with high selectivity for both, α- and β-glycosylation. Thus, using either donor **148α** or **148β** provided the corresponding glycosides **152α** or **152β**, with retention of stereochemical integrity at the anomeric center (Scheme 34a,b). The scope of the reaction was investigated with an array of alcohol nucleophiles (Scheme 34). The use of sterically hindered adamantol as a glycosyl acceptor led to moderate yields of adamantyl glycosides (≈52%–54%) along with *tert*-butyl glycoside **153β** (in the glycosylation of **148β**, Scheme 34b). Formation of the latter was explained by the presence of “departing” *t*-BuOH as a competing nucleophile in the reaction media. However, the use of excess glycosyl acceptors or the use of pyvaloyl rather than *tert*-butoxy carbonyl glycoside donors allows increased yield in the formation of adamantyl glycosides.

The starting pyranones **148** were easily accessible from furan alcohols by Achmatowicz ring-expansion [16,17] followed by stereoselective hemiacetal protection [140].

**Scheme 35:**
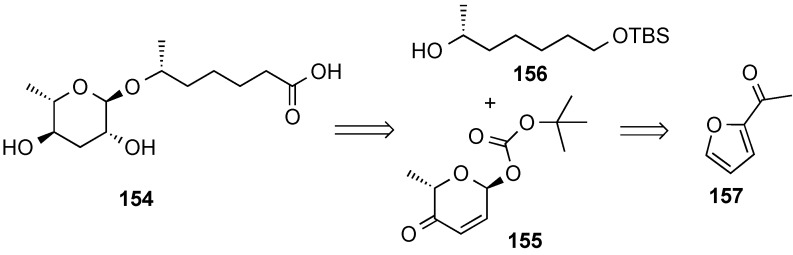
O’Doherty’s approach to daumone, **154**.

**Scheme 36:**
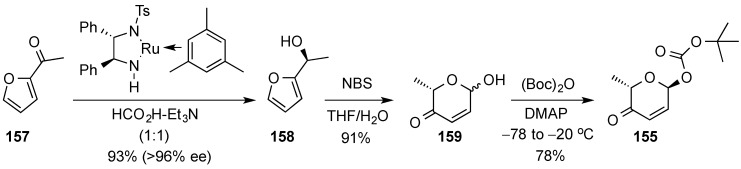
Enantioselective synthesis of α-pyranone **155**.

The diastereoselective palladium-catalyzed glycosylation was also used in the preparation of the pheromone daumone **154**, by use of pyranone **155** as the glycosyl donor (Scheme 35) [20]. The latter was prepared in enantiomerically pure form by enantioselective Noyori reduction [141] of acylfuran **157** (Scheme 36). Thus, Noyori reduction of **157** with the enantiomeric catalyst provided furan alcohol **158** in very high enantiomeric excess (93% yield, >96% ee). Ring-expanded pyranone **159** was then obtained by treatment with *N*-bromosuccinimide (NBS) in THF/H_2_O) [16,17]. Diastereoselective acylation of **159** was performed at low temperature with (Boc)_2_O provided pyranone donor **155**.

**Scheme 37:**
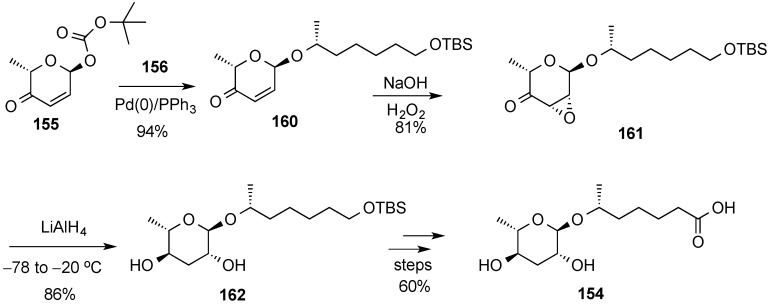
Palladium-catalyzed glycosyl coupling and processing to daumone **154**.

Palladium-catalyzed glycosylation of secondary alcohol **156** with α-pyranone **155** was carried out in CH_2_Cl_2_ to yield glycoside **160** as a single diastereomer (Scheme 37). Diastereoselective epoxidation of enone **160** to give epoxy-ketone **161** was then followed by a one-pot process involving ketone reduction and epoxide opening to give rhamnose derivative **162**. Finally, deprotection and oxidation led to daumone **154**.

O’Doherty’s group has also exploited this methodology for the synthesis of oligosaccharides, in particular 1,6-linked and 1,4-linked oligosaccharides, by way of iterative glycosylations combined with diastereoselective ketone reduction and dihydroxylation processes [142].

**Scheme 38:**
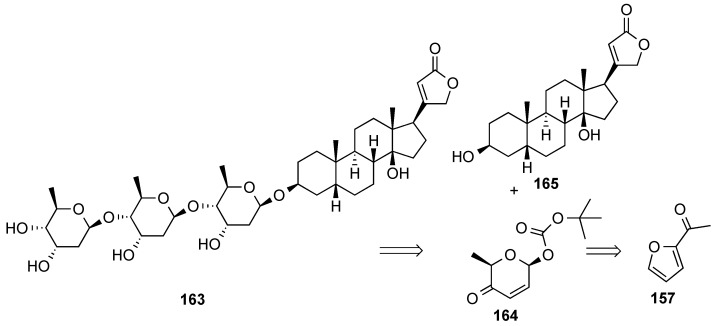
O’Doherty’s retrosynthesis of digitoxin **163**.

The iterative glycosylation protocol was also applied to the stereoselective synthesis of digitoxin **163** (Scheme 38) [143,144]. O’Doherty’s retrosynthesis for digitoxin is outlined in Scheme 38 and involved the iterative, diastereoselective, palladium catalyzed glycosylation of digitoxigenin (**165**) with pyranone **164**, which is accessible in enantiomerically pure form from acylfuran **157**.

**Scheme 39:**
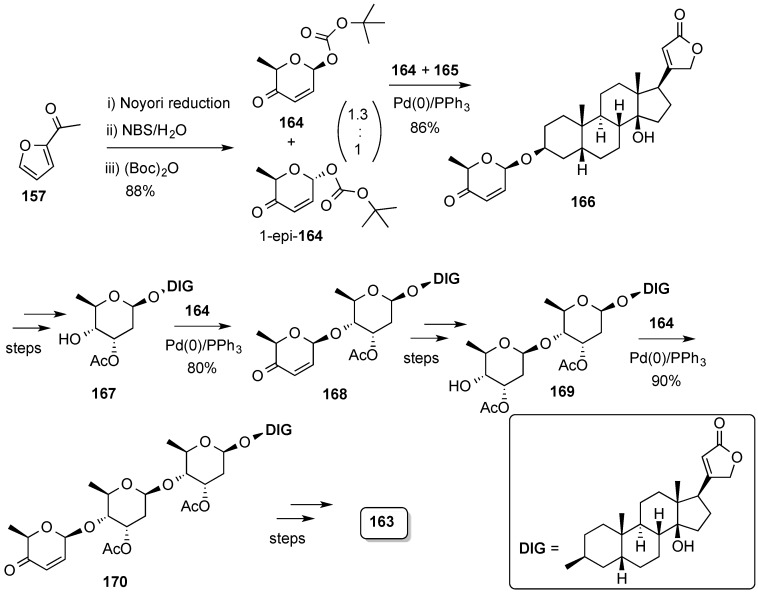
O’Doherty synthesis of digitoxin (**163**) by iterative palladium-catalyzed glycosylations.

The synthetic route started with an enantioselective Noyori reduction of acylfuran **157**, followed by the Achmatowicz ring-expansion protocol, and stereoselective anomeric Boc-formation at high temperature to obtain pyranone **164** as the major isomer (Scheme 39). Palladium-catalyzed gycosylation of digitoxigenin (**165**) with **164** produced glycoside **166**, which was processed to dihydroxy acetate **167**. Iteration of the glycosylation/pyranose functionalization processes to the di- and trisaccharides **168** and **179**, respectively, resulted in the synthesis of digitoxin, **163**.

**Figure 4:**
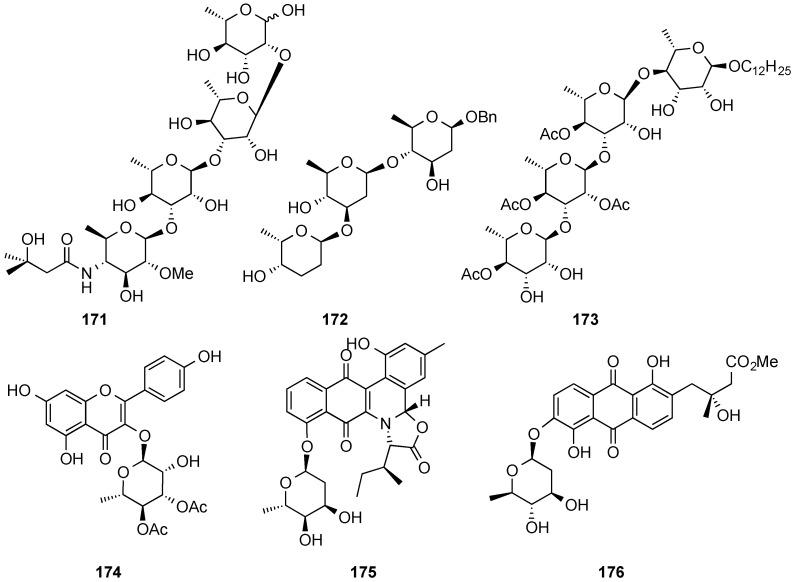
Natural products synthesized by O’Doherty’s group.

The usefulness of the protocol implemented by O’Doherty’s group from achiral furan **157** via enantioselective reduction, Achmatowicz ring-expansion, and diastereoselective (iterative) palladium-catalyzed glycosylation(s) has been further demonstrated with the successful synthesis of anthrax tetrasaccharide (**171**) [145,146,147], the trisaccharide portion of landomycin A (**172**) [148], cleistroside-2 (**173**) and several members other members of the cleistroside (tri- and tetra-rhamnosides) family [149], as well as the total syntheses of kaempferol glycoside SL101 (**174**) [150], jadomycin B (**175**) [151,152], and vineomycinone B_2_ methyl ester (**176**) [153] (Figure 4) [154].

**Scheme 40:**
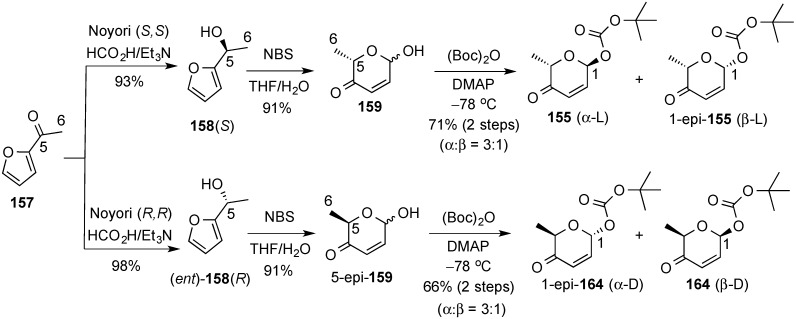
O’Doherty’s enantio- and stereo-divergent approach to d/l and α/β-pyranones **155** and **164**.

**Scheme 41:**
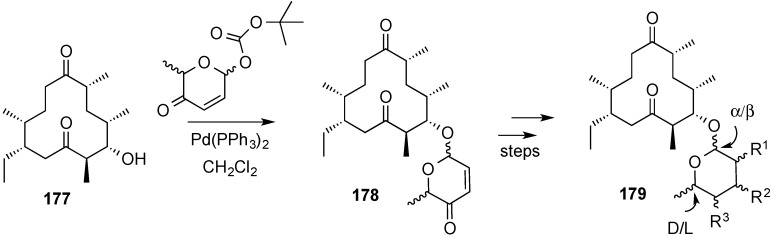
Enantio- and stereo-divergent synthesis of glycosylated methymycin analogues, **179**.

The synthetic potential of this protocol is enhanced by the flexibility of the enantioselective reduction of the acyl furans and the stereocontrol in the formation of the anomeric *tert*-butyl carbonates. Thus acyl furan **157** can be transformed, in a stereocontrolled manner in α-l, β-l, α-d or β-d *tert*-butyl carbonates **155** and **164**, respectively (Scheme 40) [155]. These derivatives were used in the preparation of a collection of 11 methymycin analogues (**179**) by stereoselective glycosylation of 10-deoxymethylnolide **177** followed by synthetic manipulations of the ensuing pyranones **178** (Scheme 41).

Related chemistry was also used in the preparation of the α-l-aculose, α-l-rhodinose, and β-d-olivose trisaccharide-component of PI-080 [156].

**Scheme 42:**
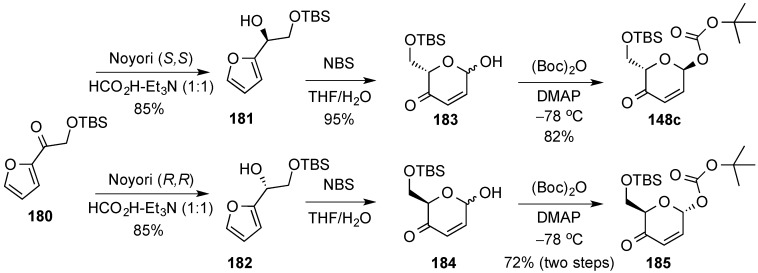
Enantiodivergent synthesis of pyranones, **148c** and **185** from acyl furan **180**.

Pyranones with oxygen substituents at the primary position, e.g., **148c**, **185**, as precursors of 6-hydroxy pyranoses, can analogously be prepared in either enantiomeric form (d/l) from oxygenated acyl furan **180** by way of enantioselective Noyori reduction (**181**, **182**), and Achmatowitcz ring-expansion (Scheme 42) [157]. A combination of d- and l-pyranones were used by O’Doherty in the *de novo* asymmetric synthesis of all-d, all-l, and d-/l- oligosaccharides (see Scheme 10, Section 3.1) [48].

**Scheme 43:**
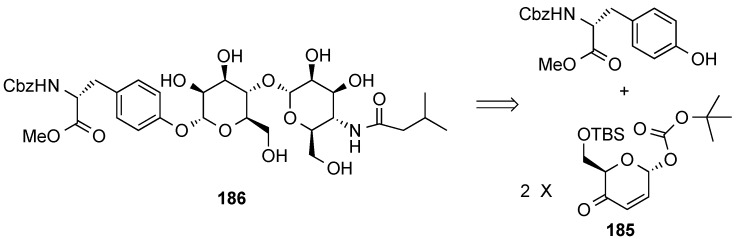
O’Doherty’s retrosynthesis of the disaccharide portion of mannopetimycin-E **186**.

Pyranone **185** has also been used in the preparation of the glycosylated tyrosine portion of mannopetimycin-E, **186** (Scheme 43) [158,159].

**Scheme 44:**
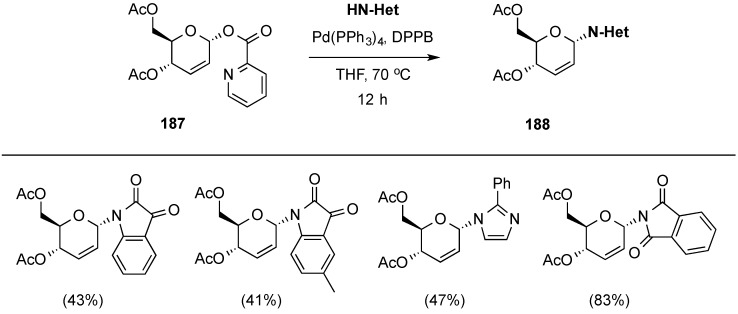
Liu and co-workers’ stereoselective palladium-catalyzed *N*-glycosylation.

Very recently, Liu and co-workers have reported the stereoselective palladium-catalyzed *N*-glycosylation of α-picoloyl 2,3-unsaturated hexopyranosides, e.g., **187**, leading to *N*-heterocyclic glycosides **188** (Scheme 44) [160]. The method, initially developed and optimized on 3-picoloyl glucals, was compatible with a variety of protecting groups on the glycosyl donor.

**Scheme 45:**
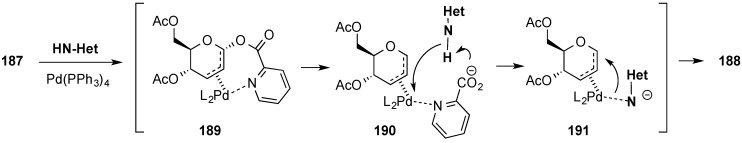
Proposed reaction mechanism for the synthesis of *N*-glycosides **188**.

Based on their results, the authors were able to propose a reaction mechanism that is outlined in Scheme 45. The pathway involved simultaneous initial palladium coordination to the double bond and to the nitrogen of the pycoloyl group at the α-face of the sugar to generate intermediate **189**. Subsequent cleavage of the picoloyl acid species yielded the π-allyl system depicted as **190**. Finally, coordination of the *N*-nucleophile to the palladium released the picoloyl acid and provided intermediate **191**, where an intramolecular nucleophilic addition takes place to yield *N*-heterocyclic glycosides **188**.

#### 3.6.2. Lewis-Acid Mediated Glycosylation of 2,3-Unsaturated Glycosyl Acetates

**Scheme 46:**
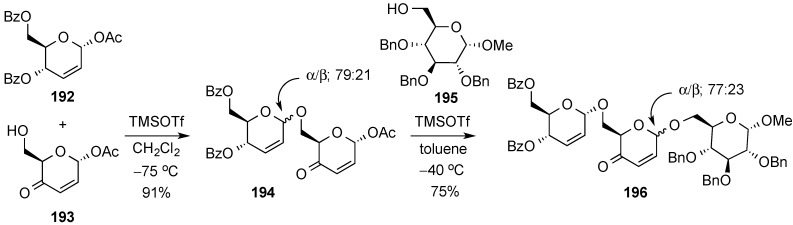
Toshima’s chemoselective glycosylation strategy to trisaccharide **196**.

Toshima and co-workers reported on the chemoselective assembly of differently substituted 2,3-unsaturated pyranoses [161,162]. Thus, 2,3-unsaturated-4-keto glycosyl acetates, e.g., **193**, were found to display lower reactivity than 2,3-unsaturated-4-hydroxy glycosyl acetates, e.g., **192**, in the presence of Lewis acids, and could therefore be used as glycosyl acceptors with the latter acting as glycosyl donors. An implementation of their strategy is outlined in Scheme 46. Accordingly, 4-keto derivative **193** was chemoselectively glycosylated with **192** by use of TMSOTf in CH_2_Cl_2_ at −78 °C, to give disaccharide **194** in fairly good yield. Subsequently, the ensuing 4-keto derivative **194** was able to act as a glycosyl donor and was used to glycosylate methyl glucoside **195**, in toluene at higher temperature, to yield trisaccharide **196** (TMSOTf, –40 °C). The observed α/β anomeric selectivity was high and in agreement with literature precedents favoring the α-anomer in each case.

#### 3.6.3. Halonium Ion-Mediated Glycosylation of 2,3-Unsaturated Allyl Glycosides

**Scheme 47:**
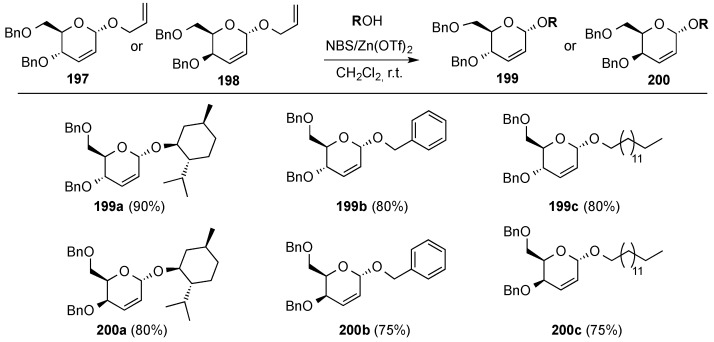
Taneja’s stereoselective α-glycosylation with allyl glycosides **197** and **198**.

Taneja and co-workers recently described the remarkable stereoselective α-glycosylation of 2,3-unsaturated allyl glycosides mediated by NBS in the presence of catalytic Zn(OTf)_2_ [163]. The method was applied to the glycosylation of a variety of alcohols with *erythro*- and *threo*- 2,3-unsaturated allyl glycosides **197** and **198**, respectively (Scheme 47). Protecting groups such as acetonide, nitro, or esters proved to be compatible with the reaction conditions.

### 3.7. Use of 2,3-Unsaturated Hexopyranoses as Chiral Complex Ligands

**Scheme 48:**
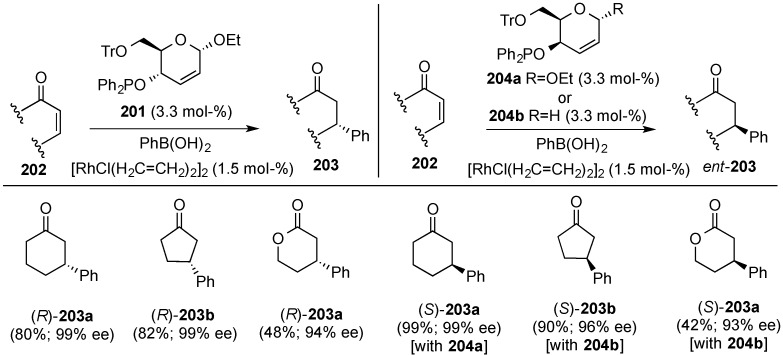
Boysen’s pair of pseudoenantiomeric carbohydrate derived phophinites **201** and **204**, in rhodium catalyzed asymmetric 1,4-addition of phenylboronic acid to unsaturated enones and enoates.

Carbohydrates have been long used as stereodifferentiating agents [164]. In this context, Boysen and co-workers reported on a phosphinite hybrid ligand **201**, based in a 2,3-unsaturated pyranoside [165]. Accordingly, phosphinite **201**, readily prepared by reaction of the corresponding unsaturated alcohol with diphenyl chlorophosphine (PPh_2_Cl, Et_3_N, THF, 70% yield), was employed in the rhodium-catalyzed 1,4-addition of boronic acids to unsaturated ketones and lactones **202**. The ensuing products, **203**, were obtained with high yields and excellent stereoselectivity when cyclic substrates were involved (Scheme 48). In a recent remarkable development, Boysen and co-workers reported that isomeric *erythro*-, *i.e.*, **201**, and threo-, *i.e.*, **204**, phosphinites, behaved as pseudo-enantiomeric olefin ligands in Rh(I)-catalyzed 1,4-additions of aryl and alkenylboronic acids to achiral enones [166]. They also extended the reaction to a variety of alkenyl and aryl boronic acids.

### 3.8. Miscellaneous

**Scheme 49:**
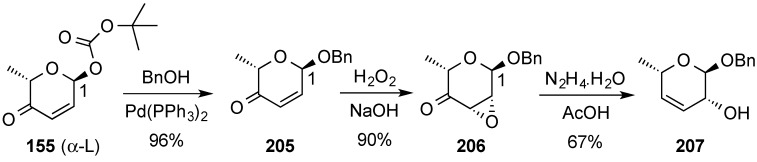
Wharton rearrangement of pyranone **205** to hex-3,4-enopyranoside **207**.

A series of synthetic transformations of *de novo* hex-2,3-enopyranose derivatives, e.g., **155**, into a variety of monosaccharide and deoxy-monosaccharide derivatives have been described by O’Doherty’s group [167]. These transformations make imaginative use of addition, oxidation, and substitution reactions performed on hex-2,3-enopyranoses and 3,4-unsaturated pyranoses, e.g., **207**, the latter readily available from the former by Wharton rearrangement (Scheme 49) [168]. Accordingly, Boc-pyranone **155** was converted by way of stereoselective Pd(0) glycosylation into α-benzyl derivative **205**, whose epoxidation under basic conditions led stereoselectively to epoxy ketone **206** [169]. Wharton rearrangement of the latter then provided benzyl hex-3,4-enopyranoside **207**.

**Scheme 50:**
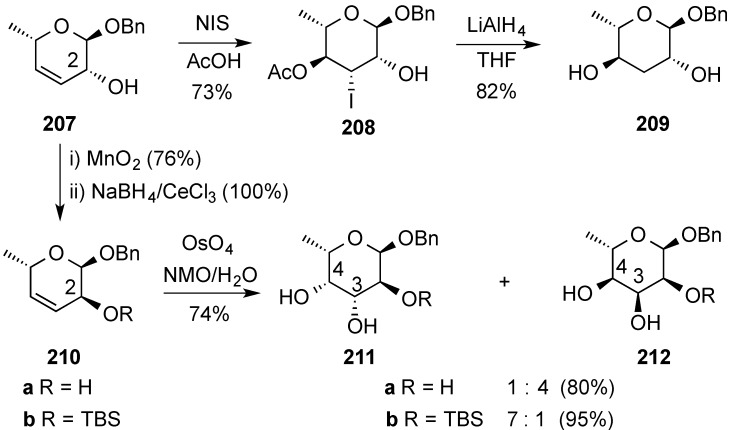
Synthetic transformations of hex-3,4-enopyranoside **207** leading to α-ascariloside **209** and α-fucosides **211**.

A synthetic route to α-ascariloside **209**, was devised by regio- and stereoselective reaction of **207** with *N*-iodosuccinimide (NIS) in acetic acid followed by LiAlH_4_ reduction of the ensuing β-acetoxy iodide **208** (Scheme 50) [169,170]. An approach to benzyl α-fucoside (**211**) from **207** was implemented via osmylation of **210** (2-epi-**207**, prepared by oxidation/reduction of **207**) (Scheme 50). It was observed that osmylation of **210** leading to fucose monosaccharides (**211**) was better carried out on 2-silyl derivative **210b**, which produced a 7:1 diastereomeric mixture favoring **211b** [211b/212b 7:1)]. Conversely, osmylation of **210a** led to diastereomeric **212a** as the major isomer [**211a/212a** 1:4)] [169].

## 4. Conclusions

Hex-2,3-enopyranosides continue to be important intermediates currently used in a variety of synthetic transformations. They are readily available by Ferrier rearrangement of commercially available glycals, although more recently the *de novo* approach to pyranones, and thence hex-2,3-enopyranosides, has positioned itself as reliable synthetic alternative for their preparation. The latter approach has the advantage of providing access to enantiomeric hex-2,3-enopyranoside pairs. The use of 2,3-unsaturated pyranosides in glycosylation has grown exponentially during the last decade, more than likely because of the success on the stereoselective Pd(0)-mediated glycosyl coupling of α- and β- pyranones.
